# Collecting near mature and immature orchid seeds for ex situ conservation: ‘in vitro collecting’ as a case study

**DOI:** 10.1186/s40529-017-0187-5

**Published:** 2017-08-08

**Authors:** Jonathan P. Kendon, Landy Rajaovelona, Helen Sandford, Rui Fang, Jake Bell, Viswambharan Sarasan

**Affiliations:** 1 Natural Capital and Plant Health, Royal Botanic Gardens Kew, Richmond, Surrey TW9 3AB UK; 2 Kew Madagascar Conservation Centre, Lot II J 131 B Ambodivoanjo-Ivandry, 101 Antananarivo, Madagascar

## Abstract

**Background:**

Lack of phenological information and efficient collecting methods are considered impediments for orchid seed collecting. This leads to opportunistic collecting as part of general seed collecting schedules that may last few weeks especially in remote areas. The study explored the feasibility of collecting near mature and immature seeds to support conservation action plans. Mature, near mature and immature seeds of orchids were collected from the wild in the Central Highlands of Madagascar (CHM). Seed capsules were collected in sterile culture medium in the wild, to prevent deterioration of seeds inside the capsule after collecting, later to be cultured under laboratory conditions.

**Results:**

Seed capsules collected by the in vitro collecting (IVC) method were kept in very good condition for up to 4 weeks before germination under in vitro conditions. Significantly faster and higher germination rate (*p* < 0.001) than mature seeds was observed in a number of taxa collected during a 3 year-long study. In some taxa even immature seeds, with no apparent sign of testa covering the embryo, germinated following IVC where mature seeds failed to germinate.

**Conclusions:**

We propose that IVC method has potential to complement conventional seed collecting by increasing the germplasm that can be used in integrated conservation action plans. Improvements can be made in developing collections for taxa from biodiversity hotspots and remote areas where collecting requires considerable resources. This method can further be used on a wider selection of plants from different geographic areas and on embryo rescue programmes for economically important plants.

**Electronic supplementary material:**

The online version of this article (doi:10.1186/s40529-017-0187-5) contains supplementary material, which is available to authorized users.

## Background

Orchids are the most complex and enigmatic group of flowering plants, with a global distribution, yet are considered to be the most threatened family. A large proportion of orchids are endemic in areas where habitat loss, illegal collecting and loss of pollinators are prevalent (Merritt et al. 2014). Due to the threats to orchids worldwide and the adverse effect on recruitment in the wild they have been included in Appendix II of the Conservation on International Trade in Endangered Species of Wild Fauna and Flora (CITES). Orchid seeds appear to be short-lived compared to crop plant seeds, even if considered desiccation tolerant (Pritchard et al. 1999; Seaton and Pritchard 2003). Seed dormancy in many plant families seriously impacts outcomes at the time seeds are sown (Merritt et al. 2005). Lack of seed information and handling practices continuously affect outcomes in restoration programmes. This includes data deficiencies pertaining to phenology of seed development and maturation for the majority of wild species (Broadhurst et al. 2008; Mortlock 2000) and this can lead to asynchronous timing of seed collection (Merritt and Dixon 2011). In orchids, irregular germination in nature as well as under in vitro conditions is a major bottleneck for conservation of rare and threatened taxa (Barsberg et al. 2013). In particular, terrestrial orchid seeds are even more difficult to work with due to quick seed maturation, dormancy-related germination issues, and desiccation sensitivity. Therefore, terrestrial orchids present significant conservation challenges.

The number of ex situ conservation facilities worldwide has grown dramatically over the years (Wyse-Jackson 2001), but have become specifically integrated to achieve objectives under national and regional conservation priorities (Maunder et al. 2001). Nevertheless, biodiversity conservation in resource poor areas of the world still faces an uncertain future. One of the key priorities of ex situ conservation is long term storage in seed banks and cryopreservation facilities which require substantial resources for capacity building. Long term storage of orchids requires cryopreservation at ultra-low temperature as a general rule because longevity under conventional seed banking conditions is not completely reliable (Merritt et al. 2014).

There are nearly 1000 orchid taxa in Madagascar, of which 85% are endemic—an exceptional area of biodiversity for orchids (Moat and Smith 2007; Tyson 2000). They are found in several phytogeographical regions with unique climatic conditions. This global biodiversity hotspot is a perfect study area to test orchids for collecting methods and assessing viability. We selected the Central Highlands of Madagascar (CHM) as our study area with over 50 species of orchids. The habitats of the study comprised inselbergs, savannah, grasslands, montane rocky scrubland and gallery forests. This area has granitic rocks, marble and quartzite with the majority of orchids being lithophytes and the rest true epiphytes and terrestrials.

In the present paper we describe methods for collecting seeds at different maturity levels from the CHM habitats using IVC methods. IVC is the method of initiating in vitro cultures from plants in the wild. The method has been used in the past for a variety of functions ranging from horticulture to conservation (Warren 1983; Alvarenga et al. 2002; Henao et al. 2002; Saldana et al. 2002; Brenes et al. 2002; Engelmann 2002; Sandoval 2002). Propagation of orchids from seeds has long been achieved through the use of capsules which are either fully matured or green, un-dehisced and near-mature. Collected dry seeds are cleaned and dried before either storage or culturing for germination. Seed viability upon storage under standard seed bank conditions is still not completely reliable for orchids (Merritt et al. 2014). In many cases, especially in temperate terrestrial orchids, higher germination rates have been achieved using green capsules than mature seeds. This method is dependent on the immediate culturing of seed after harvest from green capsules. Seeds collected from plants in the wild, spread over large swathes of land mass, from remote locations usually perish due to moisture loss and microbial contamination (unpublished results). The critical aspect of working towards developing a successful IVC protocol is the control of contamination (Pence 2005) along with maintaining the viability of the differentially matured seeds. In many cases the techniques were specifically developed for only a limited number of taxa as described in the present study. We discuss the importance of this new method to improve collecting and germination efficiency of differentially matured orchid seeds. The implications of this approach to cryopreservation and living collection development for reintroduction and assisted colonisation are discussed in detail.

## Methods

### Study area and seed collecting

**Fig. 1:**
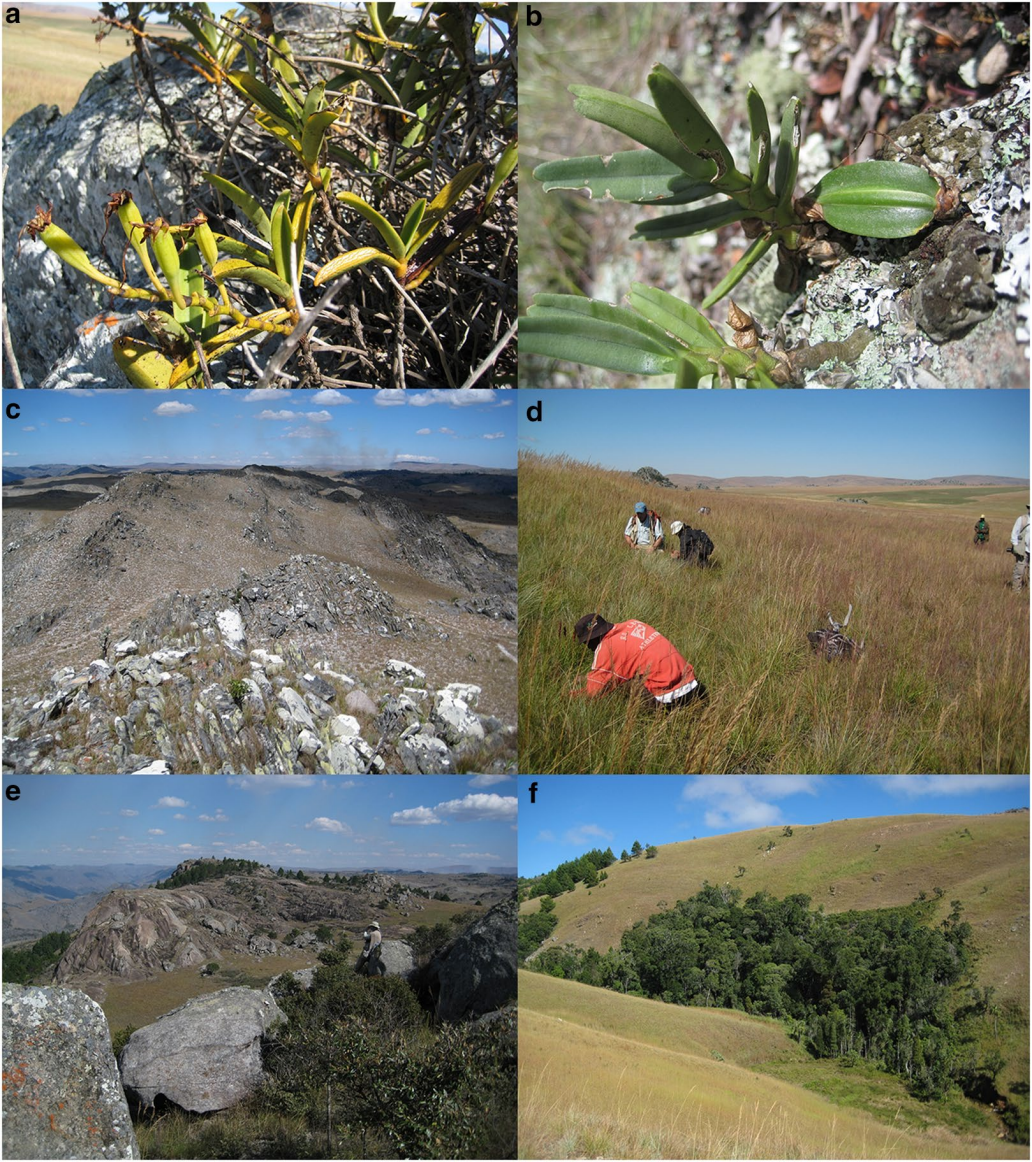
Orchids and their habitats in Itremo, Central Highlands, Madagascar. **a** *Aerangis ellisii* and **b** *Angraecum rutenbergianum* with near-mature seed capsules; **c** rocky montane grassland; **d** savannah; **e** inselberg; **f** gallery forest. Note smoke visible in tile (**c**) as a result of extensive grass fires

Studies in the field were conducted in the CHM habitats where a large number of endemic orchids are found in discontinuous populations. The orchid seed capsules were collected from plants in the wild in inselberg, savannah, montane rocky grassland, and gallery forests (Fig. 1).

Only a small number of capsules were allowed to be collected due to the fragmented nature of populations and few available capsules in individual plants. Orchids from these diverse habitats exist as epiphytes, lithophytes, and terrestrials. Different habitats and life forms contribute to the diversity of seed capsules which were available for this study. Seed capsules were categorised into five groups based on their size.

Seed capsules were considered mature when they were yielding to the touch and had yellow, red or brown colouration, and contained mature seeds. Seed capsules were collected in vitro if they contained near-mature or immature seeds. These capsules were green, firm and of similar dimensions to mature capsules. Mature capsules were cut from the plant, wrapped in paper in a sealed seed envelope and placed in a field desiccator. This consisted of a sealable plastic box containing orange silica gel. Several collections were made per species for both IVC and mature capsules, and collection was performed in an opportunistic manner in accordance with guidelines laid down by the seed conservation department, RBG Kew (Way 2003). On arrival at RBG Kew the seeds were equilibrated over a saturated solution of CaCl_2_ inside a glass desiccator, before being sealed in glass vials and stored in a fridge at 3 °C.

The following three-step procedure was employed for the IVC of seed capsules from all species collected from the wild in Itremo, CHM.

### Surface sterilisation in the field

Flower parts were removed by scalpel blade and the capsules were cleaned with moist sterile towels immediately after collection. These seed capsules were then sterilised in 0.5% (w/v) sodium dichloroisocyanurate (NaDCC) solution (Sigma Chemicals, UK) for a period depending on size, nature of seed capsule and where it was collected from (Table 1). Using results of the preliminary IVC studies, on two terrestrial orchid species at RBG Kew, the concentrations of NaDCC and PPM to be used in this study were optimised. For sizes 1–3 plastic disposable containers (150 ml volume) with screw cap lids (Sterilin, UK) were used to culture the capsules following surface sterilisation; for size 4 and 5 Dilu vials (27 ml volume, Alpha Laboratories, UK) with flip tops were used. Sterilised seed capsules were washed in one change of 0.01% NaDCC before being transferred to IVC medium containing NADCC and plant preservative mixture (PPM, Apollo Scientific, UK). The culture medium selected for Madagascar IVC was ¼ MS (Murashige and Skoog 1962) supplemented with 1% sucrose, 0.1% activated charcoal and 1% agar to which was added 0.03% (w/v) NaDCC, once the medium had cooled to around 40 °C to avoid evolution of chlorine gas, and 2 ml/l of PPM (v/v).

**Table 1:** List of taxa from Itremo, Central Highlands, Madagascar: seed capsule size and seed capsule colour 2–3 weeks after in vitro collecting and results of seed capsule sterilisation that yielded clean seeds 3 weeks after sowing

Taxon	Life form	Colour of seed pod on arrival at RBG Kew	Size group	Init ial pod sterilisat ion t ime (mins)	Response at step 1, 2 and 3 [Sterile (S)/Contaminated (C)]			Response after seed sowing
					1	2	1	
*Aerangis citrata*	*EP*	Green	4	45	S	–	–	S
*Aerangis ellisii*	*LP*	Green + brown	2	80	C	S	–	S
*Aerangis ellisii*	*LP*	Green, majority brown	2	80	C	S	–	C
*Aerangis punctata*	*EP*	Reddish tinge	4	60	S	–	–	S
*Aerangis punctata*	*EP*	Brown	4	50	C	S	–	S
*Aerangis sp.*	*LP*	Green	4	40	S	–	S	
*Aerangis sp.*	*LP*	Green	3	40	S	–	S	
*Aerangis sp.*	*LP*	Green	3	40	S	–	S	
*Aerangis sp.*	*LP*	Yellowish brown	3	60	C	C	S	S
*Angraecum calceolus*	*LP*	Green	3	50	S	–	–	S
*Angraecum magdalenae*	*LP*	Green/brown	1	90	C	S	–	S
*Aerangis protensum*	*LP, TR*	Greenish/yellow brownish	2	60	C	S	–	S
*Aerangis rutenbergianum*	*LP*	Brown	3	60	C	S	–	S
*Aerangis rutenbergianum*	*LP*	Green	4	50	S	–	–	S
*Aerangis sororium*	*LP*	Green	1	90	S	–	–	S
*Aerangis sororium*	*LP*	Brown	1	90	C	S	–	S
*Aerangis sp.*	*LP*	Greenish brown	5	40	S	–	–	S
*Bulbophyllum peyrotii*	*EP*	Brownish black	5	60	C	S	–	S
*Habenaria ambositrana*	*TR*	Green	5	60	S	–	–	S
*Oeceoclades calcarata*	*LP*	Brown	1	80	C	S	–	S
*Polystachya fusiformis*	*LP, EP*	Green	4	45	C	S	–	S
*Polystachya fusiformis*	*LP, TR*	Green/brown	5	60	S	–	–	S

Capsule size group: above 1500 mm^2^ (1), above 750 mm^2^ (2), above 300 mm^2^ (3), above 150 mm^2^ (4), below 150 mm^2^ (5)

*TR* terrestrial, *EP* epiphyte, *LP* lithophyte as found in the field. Response: *C* contaminated, *S* sterile

### Rescue of contaminated seed capsules at the CHM base

Seed capsules were observed under a stereo field microscope for contamination within 48 h and rescued if necessary using the following procedure. Capsules were carefully removed from the vial, wiped with a sterile antiseptic wipe and gently agitated in 0.5% (w/v) NaDCC plus a drop of hand soap for at least 1 h (small, little contamination) and up to 2 h (large, moderate contamination) depending on size of the capsule and level of contamination. Re-sterilised capsules were placed in vials containing IVC medium as described above.

### Final surface sterilisation before culturing at RBG Kew

Once the seed capsules arrived at RBG Kew, seeds were cultured directly into the culture medium containing MS nutrients, Phytamax (Sigma Chemicals, UK) or other appropriate media under sterile conditions depending on the species. All culture media were supplemented with 2% sucrose with pH set at 5.8 and solidified with 0.8% agar. Media were autoclaved at 121 °C for 15 min. Before pouring the media into 5 cm Petri dishes, 5 ml/l (v/v) PPM and 0.015% (w/v) NaDCC were added once the medium had cooled to around 40 °C. Seed capsules were cut open and seeds transferred to culture medium and incubated at 22 °C.

### Sonication, viability and carapace staining

This study was conducted to see the effect of seed coats on germination. Approximately 350–450 *Angraecum protensum* seeds each were suspended in water in 10 ml screw cap centrifuge tubes and sonicated for 0, 1 and 3 min in a Decon F5300b ultrasonic bath operating at a frequency of 40 kHz (Decon laboratories, Sussex UK). Sonicated seeds were assessed for viability by 2, 3, 5 triphenyl-2H-tetrazolium chloride (TTC) (VWR Leicestershire, UK) staining test (Hosomi et al. 2011). Staining to observe lignin on the carapace (covering of the embryo) was done by embedding seeds into JB-4 resin and sectioning using an ultra–microtome (Sullivan-Brown et al. 2011). Sectioned seed samples were left to dry at 60 °C and stained in Nile red (Sigma-Aldrich Co. LLC., UK) at 0.01 mg/ml and slides were left in the stain for 10 min (Yeung et al. 1996). The stain was washed off with deionised water and the sample mounted with 0.1% N-propyl-gallate (Sigma Chemicals, UK) before cover slips 24 mm × 50 mm were added. Photographs were taken with a fluorescence microscope (LEICA CTR 6000, LEICA microsystems, Germany).

### Data collection

Number of seed capsules contaminated at stage 1, stage 2 and stage 3 were recorded. For both IVC and mature seed, numbers of total full seeds within randomly selected fields under microscope (approximately 100 seeds in each field) were used to calculate the percentage of full seeds. Germination was defined as emergence of the protocorm from the testa. Germination was recorded based on the number of full seeds, discounting seeds with poorly formed or no embryos and four replicates were used. Percent of clean cultures and percent of seed germination data were analysed against the seed capsule size and geographical location of the plant. Kruskal–Wallis test was employed as a non-parametric method to look for the difference of germination rate between species because it does not assume a normal distribution of the residuals, and is applicable to comparing groups with different sample sizes (Theodorsson-Norheim 1986). The p-values of pairwise comparisons in the post hoc Dunn’s test were adjusted using Bonferroni-type adjustment to avoid type I error inflation that leads to a false positive discovery rate. Kruskal–Wallis test with post hoc Dunn’s test was performed using PMCMR package for R (Pohlert 2014) while correlation analyses used the stats package for R (R 2015).

## Results

Small numbers of capsules were collected from fragmented populations, single plants in some cases, from the CHM following the guidelines stipulated in the collecting permit from Madagascar Conservation authorities. The majority of seed capsules collected by the IVC method yielded seeds in a good state of freshness that stayed sterile inside the capsules. There was some discoloration in a small percentage of small seed capsules. Based on the size, colour and texture of the seed capsules the sterilisation regime was followed as in Table 1, which shows that almost all the seed capsules collected in Madagascar by IVC gave rise to sterile seed cultures. In total 87.5% of the capsules were sterile and the seeds were also clean after 3 months of culture. Both NaDCC and PPM were essential to suppress microbial growth at various steps of the process from wild collecting to culture of seeds under laboratory conditions.

### Major taxa studied

#### *Angraecum* spp

There are 133 identified species of *Angraecum* from Madagascar of which 125 are endemic and 15 (12%) species are found in the study area of Itremo (CHM) alone (Hermans et al. 2007). We have selected this genus to study in length about the seed behaviour of both mature and near mature seeds. This was primarily due its abundance in the granite/marble outcrops where anthropogenic pressures exist, such as fire and illegal mining.

**Fig. 2:**
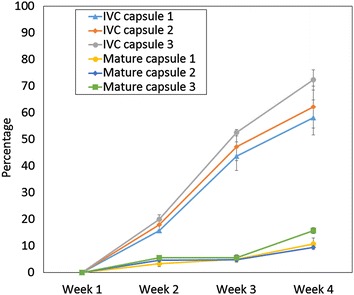
Germination of mature seeds from mature capsules versus seeds of in vitro-collected near-mature capsules of *Angraecum magdalenae*, recorded over a 4-week period. Means of four replicates ±standard error

IVC seed appeared to germinate much more quickly than mature seed. In the case of *Angraecum magdalenae* 20% of seeds germinated after 2 weeks. More than 60% average germination was achieved with IVC seeds when mature seeds showed only 20% germination after four weeks (Fig. 2).

**Fig. 3:**
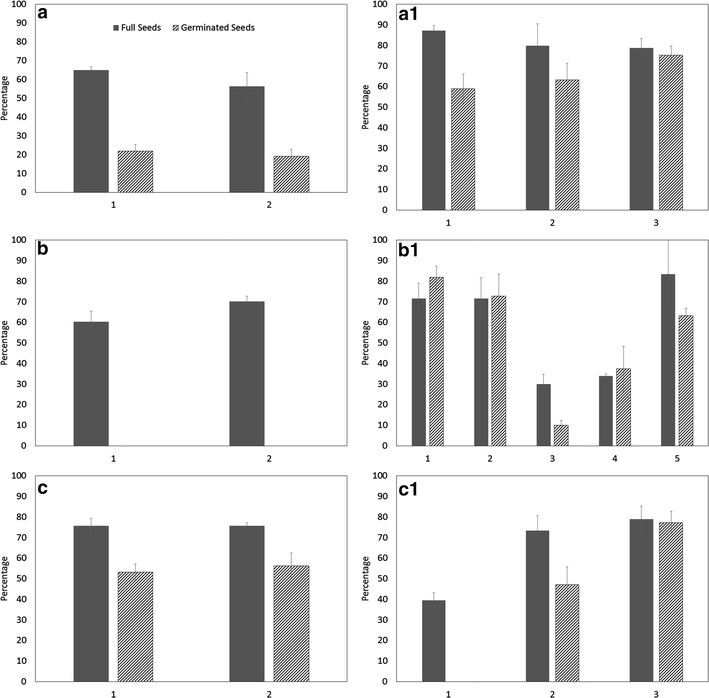
Percent full seed and seed germination after 6 months for seeds from mature capsules (*letter*) and seeds from in vitro collected near-mature capsules (*letter* + *1*) seeds. **a**, **a1** *Angraecum magdalenae*; **b**, **b1** *Angraecum calceolus*; **c**, **c1** *Angraecum rutenbergianum*; *Bar numbers* refer to mature and in vitro collected seed capsule numbers. Means of four replicates ±standard error

Mature seeds of many *Angraecum* species had a good proportion of full seeds but when compared against IVC seeds there are significant differences in germination (Fig. 3). Up to 2–3 times increase in germination was recorded when *Angraecum magdalenae* IVC seeds were germinated compared to mature seeds under in vitro culture conditions (Fig. 3). The results of the germination data of *Angraecum rutenbergianum* show the importance of collecting seed capsules at the right maturity. None of the seeds from IVC seed capsule 1 (Fig. 3) germinated because the seeds were too immature. Seeds from seed capsules 2 and 3 germinated at a reasonable percentage which is rather better than the corresponding values for mature seeds (Fig. 3).

**Fig. 4:**
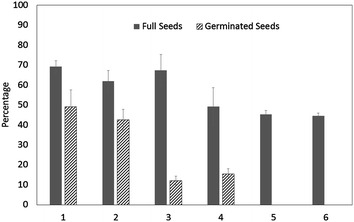
Germination percentage of mature seeds of *Angraecum protensum* originating from different seed capsules collected from Itremo, Central Highlands, Madagascar. *Each numbered bar* represents an individual capsule. Means of four replicates ±standard error

Further collections of seed capsules of *Angraecum protensum* were made and we have found wide differences in germination between seed capsules of different maturity (all seeds have coloured testa and opaque embryo but capsule colour varied from green to brown) (Fig. 4). We have observed very low germination from this species as described earlier. The histogram shows a clear difference in germination between seed capsules although most capsules had very similar percentage of full seed.

**Fig. 5:**
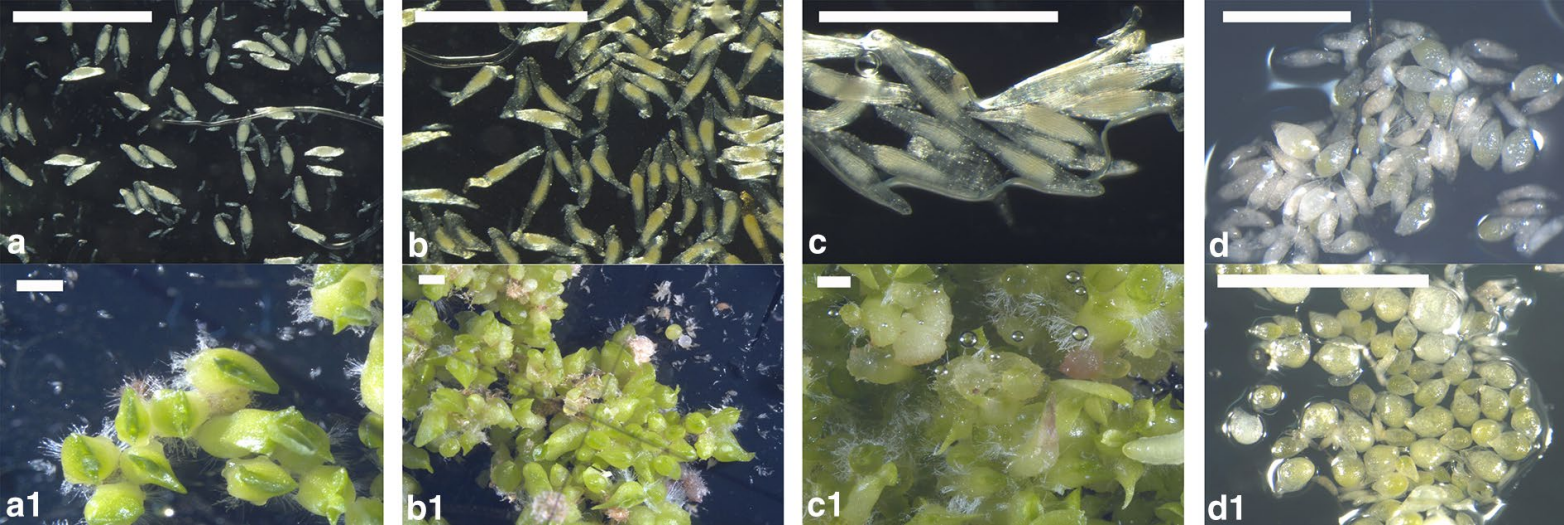
Light microscope images of in vitro collected seeds on in vitro germination medium **a** *Angraecum magdalenae*; **b** *Angraecum calceolus*; **c** *Angraecum rutenbergianum*; **d** *Angraecum rhynchoglossum* (*scale bar* 500 µm). The *lower row* (**a1**–**d1**, *scale bar* 1 mm) shows protocorms of in vitro collected seeds

Immature and near-mature seeds of *Angraecum* spp. had clear, near colourless testae in contrast to mature seeds, which have a dark brown, barely translucent testa (Fig. 5).

### Sonication, viability and carapace staining

There was an increased viability and germination, compared to the control, when seeds were sonicated for 3 min. Different sonication times (0.5–5 min) damaged the seed coat, from making perforations on the seed coat to completely opening up the seed releasing the embryo at 5 min. However, the carapace was damaged by the process of physical scarification through sonication treatments.

There were some variations in full seed percentage in both *Angraecum calceolus* and *Angraecum rutenbergianum* (Fig. 3) but not in *Angraeum protensum* (Fig. 4). Germination in mature seeds was nil (Fig. 3) in *Angraecum calceolus* but varied in both *Angraecum rutenbergianum* and *Angraecum protensum*. Seed germination was erratic in *Angraecum protensum* and for that reason we have conducted the above pilot study to understand the nature of the covering of the embryo and its effect on seed germination. The carapace was found to be a significant layer of lignin surrounding the embryo of this species. This carapace can be removed (partially or fully) by prolonged pre-treatment with different bleach concentrations and exposure times (overnight incubation in 0.1% sodium hypochlorite or sodium dichloroisocyanurate was optimum, data not included). However, we did not find a statistically significant improvement in germination when bleach was used. This could be due to harmful effects of bleach on the embryo proper.

#### *Aerangis* spp

The genus *Aerangis* has 20 species in Madagascar with 14 endemic taxa and 6 species (42%) are found in the Itremo region of CHM alone (Cribb and Hermans 2009). *Aerangis ellisii* displayed significant variation in percentage of full seeds across both IVC and conventionally collected capsules derived from different plants. This may be explained by incomplete pollination, post-pollination barriers or embryo development problems. Even freshly collected full seeds failed to germinate when the seed capsules had above 70% full seeds. With a high percentage germination through IVC this species could be a major beneficiary of this process, especially given its low abundance and sensitivity to anthropogenic fire.

**Fig. 6:**
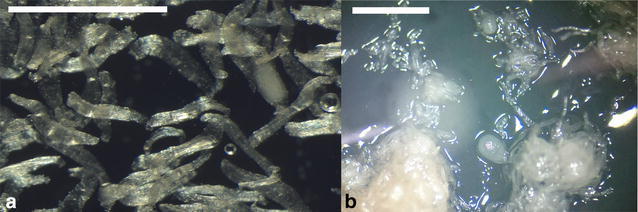
Light microscope images of **a** in vitro collected seed (*scale bar* 500 µm) in germination medium; **b** germinated protocorms (*scale bar* 1 mm) of *Aerangis ellisii*, 2 months after sowing

The maturity of *Aerangis ellisii* seeds that were collected by IVC fell into three categories i.e. (a) immature with transparent endosperm and indistinct testa, (b) immature with marginally opaque endosperm and clear testa, (c) near mature with opaque endosperm and testa. The embryos of these seeds were opaque and covered more than 2/3rd of the seed volume compared to just half in the case of the seeds with transparent embryos. Depending on the maturity of seeds germination percentage was varied. Full germination was not observed from the third category of seeds (most mature) but signs of seed germination/initiation (Fig. 6) were noticed in four out of six seed capsules ranging from 1.8 to 5.1% from thousands of seeds. Interestingly this was noticed in seed capsules with very low percentage of full seeds. On the other hand in two mature seed capsules, with more than 70% full seeds, no seeds showed any sign of germination. Most immature seeds with a testa (group 1) germinated while more mature embryos with clear testa (group 2) failed to germinate.

**Fig. 7:**
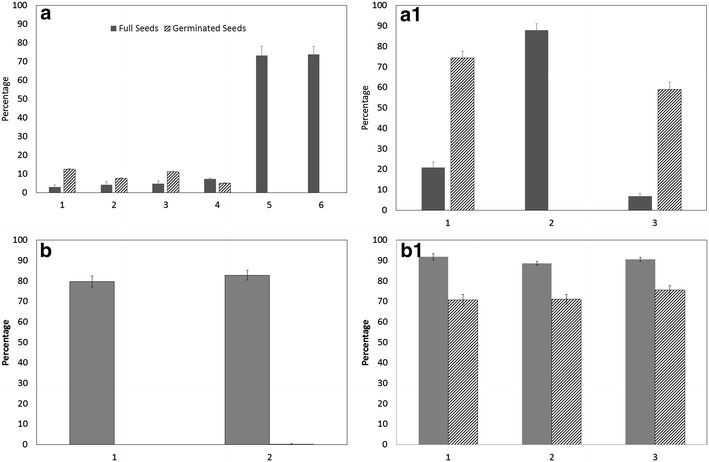
Percent full seed and germination of *Aerangis* species after 6 months. **a** *Aerangis ellisii* mature capsule; **a1** *Aerangis ellisii* in vitro collected capsule; **b** *Aerangis* sp. mature capsule; **b1** *Aerangis* sp. in vitro collected capsule. *Each numbered bar* represents a single seed capsule. Mean of four replicates ±standard error

Many capsules of *Aerangis ellisii*, both collected by IVC and as mature, had a very low percentage of full seeds (Fig. 7). *Aerangis ellisii* displayed remarkable variation between seed capsules of different maturity with mature seeds showing less than 5% germination in the majority while more than 70% germination was observed from immature seeds (IVC seeds). There were capsules in both mature and IVC which had more than 70% full seeds but germination was nil (Fig. 7).

The two mature seed capsules had 80% or more full seeds but failed to germinate except in one seed capsule where 0.3% seeds germinated (Fig. 7). However, all three seed capsules of *Aerangis* sp (yet to be identified) show exceptionally high seed germination (70% or more) from IVC seeds where nearly 90% of the seeds were full.

### Prioritisation of taxa for conservation action plans based on threat, viability and germination bottlenecks

Orchids in the study area of CHM are subjected to threats associated with anthropogenic fires, over-grazing, mining and illegal collecting. The impact of these threats on particular habitats associated with each species, together with the poor quality of mature seeds have helped us develop our assessment in Table 2. Tables 1 and 2 represent samples of capsules measured and recorded. For clarity, not all capsules collected for every species have been included. Where low numbers of individuals occur in areas of high threat levels and low mature seed germination percentage was recorded, species have been given a ‘high priority’ conservation category.

**Table 2:** Threat assessment of selected species from Itremo, Central Highlands, Madagascar, based on mature seed traits

Taxon	Life form	Geographic distribution	Percent average full seeds	Percent germination (mature)	Percent TTC viability	Conservation priority
*Aerangis ellisii*	*LP*	Core	27.7	2.3	1.9	High priority
*Aerangis* sp	*EP*	Peripheral	81.3	0.16	N/A	High priority
*Angraecum calceolus*	*LP*	Core	58.8	0	N/A	Medium priority
*Angraecum rhynchoglossum*	*EP*	Core	85.3	0	N/A	Medium priority
*Angraecum rutenbergianum*	*LP*	Core	78.5	39.4	65.2	Medium priority
*Benthamia cinnabarina*	*TR*	Core	85.6	4.7	85.7	High priority
*Benthamia rostrata*	*TR*	Core	86.4	8.2	10.5	Medium priority
*Bulbophyllum bicoloratum*	*EP*	Peripheral	88	0	0	High priority
*Bulbophyllum peyrotii*	*EP*	Core	98.4	92	87.3	Not under threat
*Disa incarnata*	*TR*	Core	60.5	8	0.3	Medium priority
*Habenaria ambositrana*	*TR*	Core	92	15.3	44.4	Medium priority
*Jumellea amplifolia*	*LP*	Peripheral	88.3	0	0	High Priority
*Liparis* sp	*TR*	Core	68.8	6.1	N/A	Medium priority
*Oeceoclades calcarata*	*LP*	Peripheral	12.6	0	65.9	High priority
*Polystachya fusiformis*	*LP*	Core	70.1	65.13	43.8	Not under threat
*Polystachya.* sp	*LP*	Core	84.8	0	0	High priority
*Polystachya.* sp	*EP*	Core	17.7	0	N/A	High priority
*Satyrum trinerve*	*TR*	Core	77.7	14.9	0	Medium priority
*Tylostigma nigrescens*	*TR*	Core	76.9	28.6	36.9	Not under threat

Categorisation: High Priority, Medium Priority and Not Under Threat currently. Geographic distribution refers to the range within core protected area or peripheral protected area, Itremo, Madagascar

*TR* terrestrial, *EP* Epiphyte, *LP* Lithophyte as found in the field, *N/A* data not available

## Discussion

**Fig. 8:**
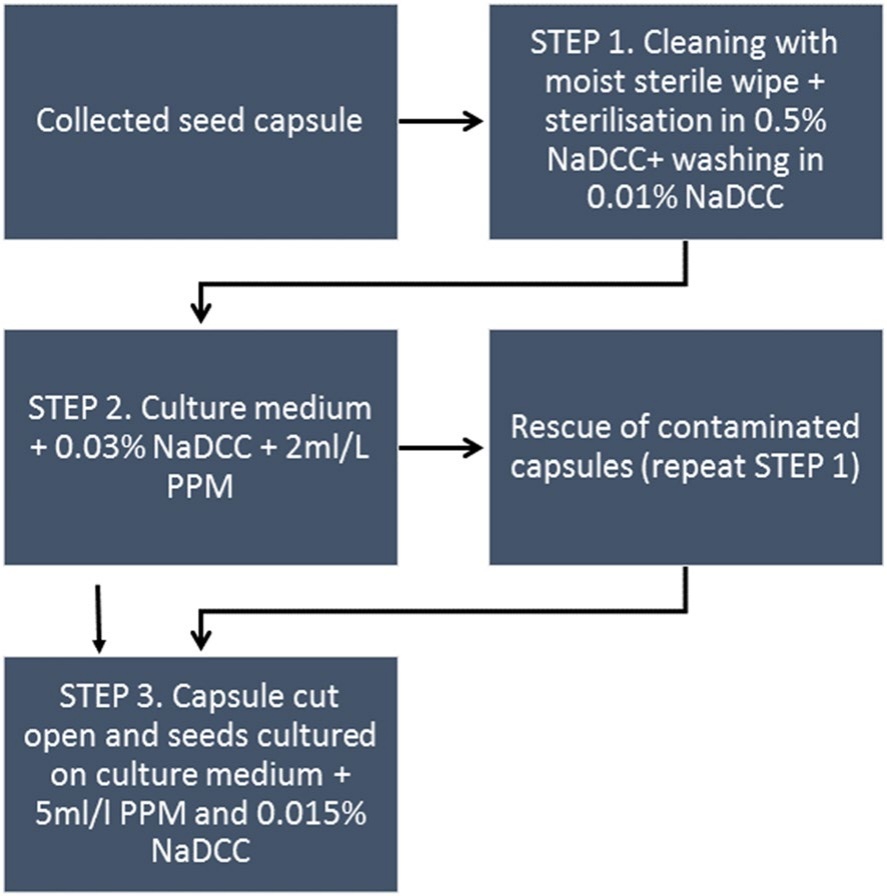
Flow chart explaining the different steps of sterilisation and culture in vitro of different types of wild collected orchid seed capsules, and final culture under laboratory conditions. Sodium dichloroisocyanurate (NaDCC) was used as sterilant and antimicrobial at different concentrations in culture while plant preservative mixture (PPM) was used at different concentrations as an antimicrobial substance

To our knowledge this is the first documented use of NaDCC (Fig. 8) as a medium additive to prevent microbial contamination during storage. Use of a lower concentration of NaDCC as a successful sterilant is due to the greater proportion of HOCl which is pH dependant (White 1986). Unlike hypochlorites NaDCC can stay stable in liquid form (Parkinson et al. 1996) due to its chemical structure and release chlorine slowly as has been reported before (Sarasan et al. 2006). This quality is useful for in vitro (Niedz and Bausher 2002) and post-harvest studies (Nicholl and Prendergast 1998) in plants. With low phytotoxicity, and damage restricted to old living tissue, NaDCC makes a suitable antimicrobial substance to be used for longer treatment as discussed in this study. Use of PPM in STEP 2 and STEP 3 was crucial to keep the capsule and seeds under sterile conditions (Fig. 8). PPM has been widely used for a range of species as an effective antimicrobial substance to reduce contamination of plan tissue cultures (Sarasan et al. 2006).

Collecting mature seeds is the generally accepted approach as seeds remain viable for a reasonable period of time for use in culture compared to less mature seeds as reported before (Stoutamire 1992). As seeds mature the seed coat becomes hydrophobic which limits water and nutrient absorption (Zhang et al. 2013) and, as reported before, in terrestrial taxa accumulation of abscisic acid (ABA) occurs as seeds mature (Kinderen 1987; Lee 2003; Lee et al. 2007). This must be the case with some of the species to prepare them to face adverse environmental conditions before they can be germinated with the help of mycorrhizal fungi in the wild. The testa of near mature seeds have living cells and are yet to acquire their hydrophobic nature. Accordingly, there were not many barriers to absorb water/nutrients from the media to germinate in vitro. In addition to the physical barrier the high endogenous ABA level in mature seeds may cause poor germination in orchids, especially terrestrial orchids (Kinderen 1987). In the cases of *Calanthe tricarinata* (Lee et al. 2007) and *Cypripedium formosanum*, the endogenous ABA level is low in immature seeds, and increases rapidly as the seeds approach maturity, coinciding with a rapid decrease in seed germination. Collecting immature seeds at 6 weeks after pollination (WAP) could give high seed permeability and low endogenous ABA, which results in improved seed germination in vitro (Lee 2003).

Mature orchid seeds showing varying germination frequencies may be demonstrating dormancy induced by inhibitory substances as reported before (Nagashima 1982a). However, immature seeds are only available during a short of window of time and need to be sown immediately because of the lack of an effective preservation method (Steele 1996; Hirano et al. 2005). It has been predicted that populations occurring in marginal habitats may be more vulnerable to repeated population crashes which can also result in a significant reduction in genetic diversity (Cozzolino et al. 2003), which may be the case in many orchids of Madagascar where only a few individuals are left in the wild.

Expanding IVC along with conventional collecting for seed banking will significantly improve the success of ex situ conservation of Madagascan orchids. Ultimately there is a great need to understand the importance of mycorrhizal fungi in epiphytes and, especially, lithophytes to improve reintroduction/restoration to support integrated conservation for meaningful outcomes in the longer term. At the moment very little is known about the mycorrhizae of lithophytes and epiphytes of Madagascan orchids except the preliminary studies conducted in our laboratory (Yokoya et al. 2015).

During embryo development in orchids the inner integument shrinks and forms a tight layer, which encloses the embryo, termed ‘carapace’ (Lee et al. 2005), mainly described in terrestrial taxa. The cuticular stain Nile Red detects this layer which may play a role in the hydrophobic nature of orchid seed and may help the seeds survive in harsh conditions. The carapace is visible in the seeds of *Angraecum protensum* as reported in terrestrial orchids (Zhang et al. 2013; Lee et al. 2005), when seeds were sectioned and stained with Nile Red. The carapace is quite prominent in lithophytic orchids, as in terrestrial taxa, and can act as a robust protective cover for the embryo. When seeds were scarified mechanically, germination percentage achieved was close to the viability percentage recorded by TTC staining. This could be the reason why IVC collected seeds performed better, with carapace under-developed, compared to fully mature seeds with well-developed carapace. In lithophytic species this may be a distinctive feature as they endure long periods of harsh conditions in a dry lithophytic habitat but detailed screening is essential to find out the trend within this group of plants. According Lee et al. (2005) the timing of seed collection outweighs the composition of the culture medium and the seed pre-treatments in a terrestrial orchid, where they have used bleach as chemical scarification. When sonication (0.5–5 min) was used, however, 3 min exposure improved seed germination by more than 30%. This treatment made perforations in the carapace which improved germination, either by reducing the hydrophobic nature of the seed, aiding moisture absorption and/or breaking the dormancy. Although this is a small study in a lithophytic orchid, the results indicate that the carapace is a barrier which protects the seeds in order to overcome adverse environmental conditions and keep seeds viable until germination can take place under favourable conditions with the help of orchid mycorrhizal fungus/fungi.

*Angraecum rutenbergianum* appeared to be a good coloniser of its habitat in the study area of Itremo. Although the species has both epiphytic and lithophytic habit, plants were found, in most cases, growing in exposed cracks and bare faces of crags and boulders. Seedling recruitment appeared to be high as large groups of seedlings could be found growing in lichen within a few metres of groups of adult plants. This would tally with a reasonable germination percentage of mature seed (Fig. 3). In contrast, *Aerangis ellisii*, which is also found as either epiphyte or lithophyte but found predominantly growing as a lithophyte in the CHM, existed in extremely small populations of large adult plants with few seedlings evident in their vicinity. Erratic pollination has been recorded in two separate studies in *Aerangis ellisii* (Nilsson and Rabakonandrianina 1988; Nilsson et al. 1992) where the pollinator activity is geographically limited. It is striking that, in both IVC and mature capsules with high percentage of full seeds, germination percentage was nil. This discovery raises further concerns for this CITES Appendix I species.

The low germination in mature seeds of *Aerangis ellisii* may be showing the consequences of inbreeding as only isolated plants were encountered in each location. This has been reported before in this species (Nilsson et al. 1988). The same kind of response was noticed in the case of mature seeds of an unidentified species of another *Aerangis*. Full seed percentage was consistent in all the capsules collected, both mature and IVC (Fig. 8), although mature seeds failed to germinate while IVC seeds germinated very well (more than 60% from all seed capsules). We have conducted further studies with small seed samples in another *Aerangis* species and noticed a similar trend. This means there may be an onset of dormancy/development of carapace layer/s as soon as the seeds start maturing. IVC can be used as a complementary tool as part of an integrated conservation action plan.

In both *Aerangis* and *Angraecum* a trend exists where IVC seeds performed far better than mature seeds and this cannot be ignored. As Madagascar is home to about 133 *Angraecum* species and more than 20 species of *Aerangis* their ex situ conservation action plans can be built around collecting seeds at immature/near mature stages to support asymbiotic models for living collection development and cryopreservation. The number of full seeds and viability of mature seeds all point to the fact that *Aerangis* is a genus which has serious conservation issues. The reported pollination behaviour of *Aerangis ellisii* (Nilsson and Rabakonandrianina 1988; Nilsson et al. 1992) explains the low full seed percentage, and the fragmentation we have found in the wild shows very little recruitment, all of which could drive the species to serious population decline in the coming decades. Detailed studies are in progress in our laboratory to understand the symbiotic relationship of this species using fungi isolated from seedlings collected from the wild.

Harding et al. (2013) reviewed the ex situ conservation of endemic flora from biodiversity hotspots, specifically using Brazil as an example and are of the opinion that ex situ conservation of endemic taxa in gene banks is often mired by information deficiency in areas of molecular genetics, seed storage and related aspects. Changing climate, population fragmentation and a lack of phenology data means reliable methods are required to collect seeds from as many species as possible to conduct trials for germination, cryopreservation and reintroduction programmes. The recent recommendation to cryopreserve orchid seeds rather than attempting to store them in conventional seed banks at −20 °C (Merritt et al. 2014) requires urgent attention. Mature good quality seed collections are a pre-requisite to achieve this system of storage. However, there are some orchid taxa which can only be collected opportunistically and in small numbers which need an alternative approach. According to Nagashima (1982b) some mature seeds have intrinsic germination problems due to accumulation of inhibitory substances and ensuing dormancy or potentially by the impermeability of the seed to a level that reduces germination following full seed maturation (Miyoshi and Mii 1988). Several methods such as sonication (Miyoshi and Mii 1988), scarification by chemicals (Mweetwa et al. 2008), and chilling (Kauth et al. 2011) have been reported to improve germination by degrading the physical barrier. According to Rasmussen (1995) the alternative to bypass this bottleneck is by sowing immature seeds without any pre-treatment. For example, seeds of *Cymbidium goeringii* showed higher germination when they were harvested close to the completion of embryogenesis but not at seed maturation Nagashima (1982b). As seeds are available only during a specific window of time they should be collected at the right time and sown immediately. When an in vitro laboratory is remotely located and access to facilities is limited it is difficult to collect plant material at the right maturity, therefore, IVC has applications to circumvent this bottleneck.

Statistical analysis showed that the tribe Vandeae, comprising the genera *Aerangis, Angraecum, Jumellea* and *Polystachya* had significantly higher germination of seeds collected through IVC than conventionally collected mature seeds. This suggests a trend in seed behaviour in this taxon. In other tribes (here including Podochileae, Cymbidieae and Orchideae), there was no significant difference between the germination of mature versus IVC seeds. The majority of epiphytic species from Madagascar can be collected by the standard collecting method as we have noticed in several species of *Bulbophyllum* and *Polystachya*. This shows that in these taxa, for seed that is viable, IVC does not lead to deterioration of seed and can complement conventional collecting as part of collecting for conventional seed banking.

Due to restricted availability of orchid capsules from the wild the numbers of capsules obtained for each species varied, as seen in Figs. 3, 4 and 7. Only mature seeds were available to collect in the case of *Angraecum protensum*. In order to justify the improved orchid seed germination by IVC method a data set consisting of full seed ratio and germination rate for collections of 6 species was subject to Kruskal–Wallis one-way analysis of variance. Comparing with mature seed the collections by IVC method promoted the germination rate of overall collection of six species in the present study with high significance (*p* < 0.001), No difference of germination rate observed between species (*p* = 0.29). The unverified species *Aerangis* sp. showed significantly higher full seed ratio than *Aerangis ellisii*. (*p* < 0.01) (Kruskal–Wallis test results available in Additional file 1). Pearson’s product-moment correlation test on the total 37 observations in the overall data set revealed a weak positive linear relationship (*r* = 0.35) between full seed ratio and germination rate of those collected seed (*p* < 0.05; Additional file 1).

One of the principal threats in the CHM habitats is fire (Hermans et al. 2007; Whitman et al. 2011) and a number of lithophytic orchids may be more prone to threats of extinction from the studied habitats than terrestrial orchids. This group of vulnerable taxa from the CHM should be prioritised and targeted for ex situ collections with IVC as a potential tool to complement conventional seed collecting.

## Conclusions

There are several impediments to work on species of high conservation value especially in small islands and biodiversity hotspot countries. The main problems are remoteness of the area for international teams who collaborate with local partners to access plants in the wild, few seed samples to collect, and in particular only limited information available on the phenology of seed capsule development and maturity. Seed storage of recalcitrant crop wild relatives, which underpins food security in the coming decades, and threatened plants (Li and Pritchard 2009) requires high quality materials. Collecting of recalcitrant seeds for large scale storage programmes require tangible sterilisation models to keep the embryonic axis/embryos in good condition until they can be processed in the lab. Our method using NaDCC offers great potential to work on storage, especially cryopreservation. Berjak et al. (2014) demonstrated the efficiency of NaDCC to keep materials in good condition before cryostorage.

For orchids our work done in the Central Highlands of Madagascar points to the fact that large genera such as *Angraecum* and *Aerangis* are facing serious threats to their existence due to low seed set, poor quality of seeds, and intrinsic poor recruitment in addition to the well-documented problems of habitat loss and climate change. As terrestrial taxa have specific maturity periods for their peak viability IVC offers great potential for collecting seeds at the right maturity for species where phenological data is available.

The method described here has the potential to aid species with recalcitrant seeds, and vegetative materials from critically endangered species. We believe our findings also have the potential to harvest seeds straight from the plant to growth medium without creating a major shock to the physiology of the seed which is essential for both applied research in groups of high conservation and crop value, and for experimental studies.

## Additional file

**Additional file 1.** Kruskal-Wallis rank sum test.
