# Supplementary material for: Collecting near mature and immature orchid seeds for ex situ conservation: ‘in vitro collecting’ as a case study
Source: Bot Stud. 2017 Aug 8;58:34. doi: 10.1186/s40529-017-0187-5 (PMC5548706; doi:10.1186/s40529-017-0187-5)
Supplement: Supplementary file 1 — Additional file 1. Kruskal-Wallis rank sum test. [file 40529_2017_187_MOESM1_ESM.docx]

**Supporting Material of Kruskal-Wallis test results**

| **SPP name** | **SPP code** |
| --- | --- |
| Angraecum magdalenae | 1 |
| Angraecum calceolus | 2 |
| Angraecum rutenbergianum | 3 |
| Angraecum protensum | 4 |
| Aerangis ellisii | 5 |
| Aerangis sp | 6 |

**1. Difference of Full-seed rate between species, regardless of maturity of seed.**

Kruskal-Wallis rank sum test

data: Fullseed by SPP

Kruskal-Wallis chi-squared = 16.987, df = 5, p-value = **0.004524**

Pairwise comparisons using Dunn's-test for multiple

comparisons of independent samples

data: Fullseed and SPP

1 2 3 4 5

2 1.0000 - - - -

3 1.0000 1.0000 - - -

4 1.0000 1.0000 1.0000 - -

5 0.5584 1.0000 1.0000 1.0000 -

6 1.0000 0.1288 1.0000 **0.0348 0.0028**

P value adjustment method: bonferroni

**2. Difference of Germination rate between species, regardless of maturity of seed.**

Kruskal-Wallis rank sum test

data: Germ by SPP

Kruskal-Wallis chi-squared = 6.217, df = 5, p-value = 0.2857

**3. Difference of Germination rate between Maturities, regardless of species of seed.**

Kruskal-Wallis rank sum test

data: Germ by Mature

Kruskal-Wallis chi-squared = 13.568, df = 1, p-value = **0.0002301**

**Supporting Material of Correlation test results**

Pearson's product-moment correlation

t = 2.2148, df = 35, p-value = 0.03338

alternative hypothesis: true correlation is not equal to 0

95 percent confidence interval:

0.02999728 0.60580564

sample estimates:

cor 0.3506085
